# Supplementary material for: Reconciling Mining with the Conservation of Cave Biodiversity: A Quantitative Baseline to Help Establish Conservation Priorities
Source: PLoS One. 2016 Dec 20;11(12):e0168348. doi: 10.1371/journal.pone.0168348 (PMC5173368; doi:10.1371/journal.pone.0168348)
Supplement: S1 Dataset — (ZIP) [file pone.0168348.s002.zip › Taxa/Serra Norte/SN_2013/N4E/Lista N4E-30.pdf]

CAVIDADE N4E-0030

Classe	Ordem	Fam/Outros	Gên/Outros	Espécie	S-U	U-S	Zon.
Arachnida	Acari	Labidostommatidae		sp.53	X		E
Arachnida	Araneae	Araneidae		jovem		X	E
Arachnida	Araneae	Barychelidae		jovem	1		E
Arachnida	Araneae	Corinnidae		jovem	1		E
Arachnida	Araneae	Ctenidae		jovem		1	E
Arachnida	Araneae	Filistatidae		jovem	X		E
Arachnida	Araneae	Mysmenidae	*Mysmenopsis*	sp.1		X	E
Arachnida	Araneae	Ochyroceratidae		jovem	X		E
Arachnida	Araneae	Pholcidae	*Mesabolivar*	aff. *cambridgei*	X		E
Arachnida	Araneae	Pholcidae	*Mesabolivar*	sp.		X	E
Arachnida	Araneae	Pholcidae	*Mesabolivar*	sp.1	X		E
Arachnida	Araneae	Pholcidae	*Metagonia*	sp.		X	E
Arachnida	Araneae	Pholcidae	Ninetinae	sp.1	X		E
Arachnida	Araneae	Salticidae		jovem	X	X	E
Arachnida	Araneae	Scytodidae	*Scytodes*	*eleonorae*	1	1	E
Arachnida	Araneae	Tetragnathidae		jovem		X	E
Arachnida	Araneae	Theridiidae	*Theridion*	sp.1	X	X	E
Arachnida	Araneae	Theridiosomatidae	*Plato*	sp.	X	X	E
Arachnida	Araneae	Thomisidae		jovem	X		E
Arachnida	Opiliones	Stygnidae	*Protimesius*	*laevis*	2		E
Chilopoda	Scolopendromorpha	Scolopocryptopidae	*Newportia*	sp.		X	E
Chilopoda	Scutigeromorpha	Pselliodidae	*Sphendononema*	*guildingii*	1	2	E
Diplopoda	Polydesmida	Chelodesmidae		sp.		X	E
Entognatha	Collembola	Paronellidae		sp.1	X	x	E
Entognatha	Diplura	Campodeidae		sp.1		X	E
Insecta	Blattodea			jovem	2	X	E
Insecta	Coleoptera	Lampyridae		jovem		X	E
Insecta	Coleoptera	Scarabaeidae		sp.		X	E
Insecta	Diptera	Bibionidae		sp.		X	E
Insecta	Diptera	Cecidomyiidae		sp.		X	E
Insecta	Diptera	Dolichopodidae		sp.	X		E
Insecta	Embioptera			sp.1	X		E
Insecta	Heteroptera	Nabidae	*Arachnocoris*	jovem	X		E
Insecta	Heteroptera	Reduviidae	Emesinae	sp.	1	X	E
Insecta	Heteroptera	Reduviidae	Reduviinae	sp.	2		E
Insecta	Homoptera	Cixiidae		jovem	X	X	E
Insecta	Hymenoptera	Formicidae	*Camponotus*	sp.1	X	X	E
Insecta	Hymenoptera	Formicidae	*Dolichoderus*	aff. *bispinosus*	X	X	E
Insecta	Hymenoptera	Formicidae	*Odontomachus*	*haematodus*		X	E
Insecta	Hymenoptera	Formicidae	*Paratrechina*	sp.		X	E
Insecta	Hymenoptera	Formicidae	*Pheidole*	sp.1	X		E
Insecta	Hymenoptera	Formicidae	*Pheidole*	sp.2	X		E
Insecta	Isoptera	Termitidae	*Nasutitermes*	sp.	X	X	E
Insecta	Lepidoptera	Tineoidea		sp.2	X	X	E
Insecta	Orthoptera	Phalangopsidae	*Phalangopsis*	sp.	X		E
Insecta	Plecoptera	Gripopterygidae		jovem	1		E
Insecta	Psocoptera	Epipsocidae	*Epipsocus*	sp.2	X		E
Insecta	Psocoptera	Manicapsocidae	*Nothoentomum*	sp.1	X		E
Insecta	Psocoptera			jovem	X	X	E
Gastropoda	Pulmonata	Subulinidae	*Allopeas*	sp.		X	E
Amphibia	Anura	Strabomantidae	*Pristimantis*	cf. *fenestratus*	8		E
Amphibia	Anura			sp.		1	E
Mammalia	Chiroptera	Emballonuridae	*Peropteryx*	*kappleri*	4		E
Mammalia	Chiroptera			sp.		5	E
